# A review of strategies used to identify transposition events in plant genomes

**DOI:** 10.3389/fpls.2022.1080993

**Published:** 2022-12-01

**Authors:** Marko Bajus, Alicja Macko-Podgórni, Dariusz Grzebelus, Miroslav Baránek

**Affiliations:** ^1^ Mendeleum—Institute of Genetics, Faculty of Horticulture, Mendel University in Brno, Lednice, Czechia; ^2^ Department of Plant Biology and Biotechnology, Faculty of Biotechnology and Horticulture, University of Agriculture in Krakow, Kraków, Poland

**Keywords:** transposable elements, transposon mobilization, course of transposition, detection methods, eccDNA, bioinformatics tools

## Abstract

Transposable elements (TEs) were initially considered redundant and dubbed ‘junk DNA’. However, more recently they were recognized as an essential element of genome plasticity. In nature, they frequently become active upon exposition of the host to stress conditions. Even though most transposition events are neutral or even deleterious, occasionally they may happen to be beneficial, resulting in genetic novelty providing better fitness to the host. Hence, TE mobilization may promote adaptability and, in the long run, act as a significant evolutionary force. There are many examples of TE insertions resulting in increased tolerance to stresses or in novel features of crops which are appealing to the consumer. Possibly, TE-driven *de novo* variability could be utilized for crop improvement. However, in order to systematically study the mechanisms of TE/host interactions, it is necessary to have suitable tools to globally monitor any ongoing TE mobilization. With the development of novel potent technologies, new high-throughput strategies for studying TE dynamics are emerging. Here, we present currently available methods applied to monitor the activity of TEs in plants. We divide them on the basis of their operational principles, the position of target molecules in the process of transposition and their ability to capture real cases of actively transposing elements. Their possible theoretical and practical drawbacks are also discussed. Finally, conceivable strategies and combinations of methods resulting in an improved performance are proposed.

## Introduction

Transposable elements (TEs) were found and described in the early 1950s by Barbara McClintock in maize, as entities causing chromosome breakage, with breaking points capable of changing their chromosomal positions (Mc Clintock, 1950). The importance of her observation has eventually been recognized as fundamental and finally, more than 30 years after publishing her seminal paper, McClintock was awarded the Nobel prize (Ravindran, 2012).

TEs are abundant structural genome components inhabiting genomes throughout the course of life evolution (Chuong et al., 2017). Initially, TEs were considered unnecessary or even harmful components of the genome (Sotero-Caio et al., 2017). At present, it is commonly accepted that their interactions with the host genome are far more complex and still not fully understood. In plants, TEs are important drivers of genome evolution, propelling phenotypic variability in the course of crop domestication and improvement. Their representation in plant genomes varies, ranging from approximately 20% in small genomes, such as Arabidopsis to more than 80% in maize (Kim, 2017).

TEs are divided into two classes, according to their mechanism of transposition: Class I (retrotransposons) and Class II (DNA transposons). Retrotransposons use an RNA intermediate to be copied and subsequently inserted as a novel copy at a new position in the genome, which results in an increase of their copy numbers (Feschotte et al., 2002). Retrotransposons are further subdivided into those harboring long terminal repeats (long terminal repeat retrotransposons, LTR-RTs) and non-LTR retrotransposons, including Long Interspersed Nuclear Elements (LINEs) and Short Interspersed Nuclear Elements (SINEs). LTR-RTs are predominant in the TE landscape of plant genomes (Satheesh et al., 2021). In contrast, most DNA transposons physically excise and reinsert (a ‘cut and paste’ mechanism), while those classified as Helitrons utilize a ‘rolling circle’ mechanism for their transposition. Thus, transposition of Class II TEs does not involve any RNA intermediate. DNA transposons are widespread and active across many bacterial, archaeal and eukaryotic species, while their activity in mammals is low (Rodriguez-Terrones and Torres-Padilla, 2018). The distribution of TEs in plant genomes has been reviewed in more detail by Sahebi et al. (2018).

Most successful TE mobilization events are neutral or even deleterious to the host. They can cause changes in the pattern of gene expression and alter gene function by up- or down-regulating adjacent genes following insertion into promoter regions, introns, exons or downstream regions (Makarevitch et al., 2015; Deneweth et al., 2022). Also, they may become a source of small interfering RNAs (siRNAs) (Piriyapongsa and Jordan, 2008; Gill et al., 2021). In order to protect integrity of the host genome, TEs are silenced and the state is epigenetically heritable (Fultz et al., 2015). In general, *de novo* silencing of active TE involves DNA methylation and repressive modifications of histones. These epigenetic marks are maintained across subsequent mitotic divisions and transmitted from generation to generation. Importantly, precise mechanisms resulting in TE inactivation depend on the location of a TE copy in the genomic context (Sigman and Slotkin, 2016)

**Figure 1:**
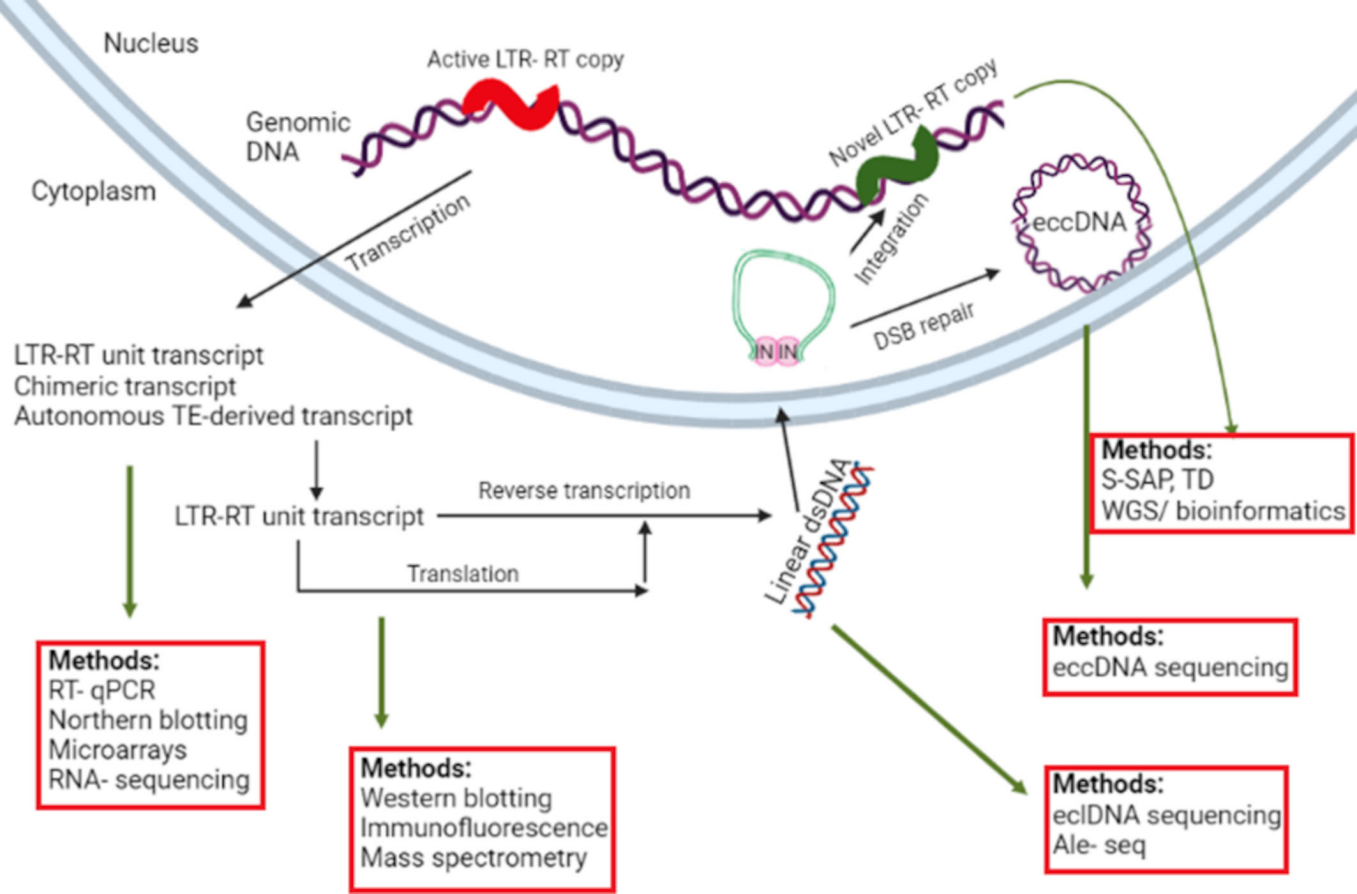
An overview of target molecules generated in the course of LTR-RT transposition and methods suitable for their detection. The meaning of individual abbreviations is as follows: LTR-RT, Long Terminal Repeat Retrotransposon; RT-qPCR, Reverse Transcription – quantitative PCR; IN, integrase; DSB, Double Strand Break; eclDNA, Extrachromosomal Linear DNA; eccDNA, extrachromosomal circular DNA; S-SAP, Sequence-Specific Amplification Polymorphism; TD, transposon display; WGS, Whole Genome Sequencing; ALE-Seq, Amplification of LTR of eclDNAs followed by Sequencing.

In order to recognize TEs showing ongoing activity, it is necessary to use tools targeting one of the molecules produced in the course of mobilization, i.e. RNA transcripts, extrachromosomal linear DNA (eclDNA), extrachromosomal circular DNA (eccDNA), small RNA or TE-encoded proteins (**Figure 1**). It is also important to monitor whether mobilized copies are competent to successfully reintegrate with the host genome to produce novel insertion sites.

The approach used by B. McClintock can be viewed as the first method of monitoring TE activity, as she observed that Ac as an activator autonomous TE mobilized non-autonomous Ds elements resulting in chromatid breakage. Fortunately, we have come a long way since then, and new possibilities and approaches are constantly emerging. The subject of the review is to summarize methods used for the analysis of TE activity and to discuss their advantages and specific applications. Special attention is paid to the LTR-RTs, which are considered the most abundant TEs in plant genomes (Deniz et al., 2019). The described methods are divided on the basis of their operational principles, the position of target molecules in the process of transposition and their ability to capture real cases of actively transposing elements. Their possible theoretical and practical drawbacks are also discussed. Finally, conceivable strategies and combinations of methods resulting in an improved performance are proposed.

## Detection of TE-derived transcripts

As LTR-RTs require the formation of an RNA intermediate, it is the first target usable for the evaluation of their activity. Generally, LTR-RT-derived RNAs can be identified using tools similar to those used for monitoring gene expression, i.e. techniques based on nucleic acid hybridization (northern blotting, microarrays), PCR (RT-qPCR), or transcriptome sequencing (RNA-seq).

Historically, northern blotting was used as the first method of choice (Manninen and Schulman, 1993; Meyer et al., 1994; Pozueta-Romero et al., 1995). With the development of new technologies, its significance gradually declined due to the complexity of protocols and necessity to ensure high amounts of input RNA. Subsequently, methods based on RT-qPCR started to be utilized to monitor TE activity in plants (Marcon et al., 2015; Paz et al., 2015; Jiang et al., 2016; Voronova, 2019; Usai et al., 2020). An important limitation of RT-qPCR is that it targets individual copies or TE families grouping very similar copies and specificity is provided by primers used for qPCR. Hence, the assay requires prior knowledge about propensity of the studied copy to be mobilized. On the other hand, it may be problematic to design specific primers to investigate TEs from different families (Morillon et al., 2002). Another limitation is the fact that the target sequence may include nucleotide substitutions and/or indels in transcripts produced from different copies. In such case, northern blotting seems to be a good complementary method, as it may reveal the size distribution of TE-derived transcripts, including full length TEs (Böhrer et al., 2020).

A global analysis of TE-derived transcripts can be produced with microarrays (Picault et al., 2009; Rocheta et al., 2016). Comprehensive information about the whole spectrum of actively transcribed TEs can also be captured by RNA-seq based on massive parallel DNA sequencing technologies (Gürkök, 2017; Oberlin et al., 2017; Qiu and Ungerer, 2018; Vangelisti et al., 2019; Jiménez-Ruiz et al., 2020; Kirov et al., 2020). RNA-seq data have been utilized and interpreted differently in reports aiming at the description of global activity of TEs. While some reports simply presented a spectrum of TEs captured in RNA-seq reads (Gürkök, 2017; Jiménez-Ruiz et al., 2020), in other reports, especially those concerning plant species for which high quality reference genomes were available, TE-derived transcripts were mapped to the reference genome assembly (Li et al., 2010; Hollister et al., 2011; Valdebenito-Maturana and Riadi, 2018). However, owing to the fact that some TE families comprise numerous copies and the evolutionary relationships among TE families can be complex, interpretation of the RNA-seq data remains challenging. Different strategies have been implemented, solely or in combination, to confirm TE expression from RNA-seq data, i.e. mapping TE-derived reads to a reference genome, a TE pseudogenome and a model transcriptome (Lanciano and Cristofari, 2020). Precision of the mapping process can be significantly improved by using longer reads provided by PacBio or Oxford Nanopore technologies (Sexton and Han, 2019). When using them it is much easier to predict if the sequenced TE-derived transcript has a potential to complete its full life cycle, or vice versa, whether it does not contain signs of inactive forms such as chimeric transcripts. Available bioinformatic tools and techniques for TE mapping to reference genomes were recently reviewed by O'Neill et al. (2020).

In general, with respect to all TE-derived transcript targeting techniques, it is necessary to be aware that there are issues that can impact clarity of results when the primary interest is to investigate only actively transposing TEs. It is because a significant share of TEs is transcribed by PolII and processed into 21~24 nt siRNA, involved in epigenetic silencing of TEs (Tang et al., 2022). Moreover, stress-dependent genome demethylation (Pandey et al., 2017; Liang et al., 2019) may result in increased expression of TEs. Also, transcripts containing sequences derived from TEs may also include chimeric transcripts containing both TE and genic fragments, e.g. those resulting from the initiation of transcription from a TE promoter or from exonization of intronic TE insertions. Such transcripts are obviously not an indication of ongoing transposition activity, but still they can be abundant in RNA samples. Besides, active post-transcriptional suppression mechanisms by TE-derived sequences was also described (Fultz et al., 2015). The above-described drawbacks and the fact that transcription is only an initial step in the process of transposition suggest that monitoring TE-derived transcripts is not an optimal strategy aiming at the identification of TEs capable of completing new insertion. There is a serious risk of misinterpretations and incorrect conclusions deeply discussed also by Deininger et al. (2017). However, expression-based assays can be used to support results concerning TE mobility produced by using other approaches.

## Detection of TE-encoded proteins

One of the possible manifestations of TE mobilization is translation of TE-encoded proteins constituting an essential transposition machinery. Thus, theoretically such proteins can also be used for monitoring an ongoing process of TE mobilization. It is necessary to emphasize that some types of TEs, e.g. SINEs or MITEs, referred to as non-autonomous, do not encode any proteins and utilize transposition machinery provided by their autonomous counterparts, LINEs and related DNA transposons, respectively. Historically, proteomic studies related to TE activity were based on western blotting. Western blot is an analytical technique used to detect a specific protein in a mixture of all proteins extracted from a tissue sample. Thus, TE mobilization-related experiments focus on a limited group of TE-derived proteins, such as transposases (Torres et al., 2013). The advantage of western blotting is that it can reveal events where internal mutations within coding regions of a TE prevent protein translation and subsequently hamper TE transposition. Such cases remained unrevealed by the analysis of TE-derived transcripts. Drawbacks of western blotting include limited availability and sensitivity of reagents, potential nonspecific activity of antibodies between related families of TEs, and necessity to produce large quantities of the starting material.

One of the most promising approaches for proteomic analysis is the application of methods based on mass spectrometry (MS) that may provide broad-spectrum results. Generally, MS is used to determine the mass of particles in order to determine the elemental composition and chemical structure of molecules, including complex substances, such as peptides. In the case of peptide analysis, combination of liquid chromatography (LC) with MS (LC-MS or LC-MS/MS), allowing for broad-spectrum analyses even down to the level of their amino acid sequences, are the most frequently used techniques. Obtained sequences can subsequently be evaluated with respect to the presence and the type of TE-derived proteins in analysed samples (Maringer et al., 2017). For example, Vuong et al. (2019) used MS to identify proteins of human TEs belonging to the L1 family of LINEs. In turn, Wang et al. (2008) used LC-MS/MS to study proteins activated by the moss *Physcomitrella patens* upon high salinity stress, revealing TE-derived proteins as being differentially expressed. Matrix Assisted Laser Desorption Ionization - Time of Flight (MALDI-TOF-TOF) combined with MS was also used to reveal proteomic background of sporadic flowering in bamboo species, suggesting a direct relationship of TE activation and the induction of flowering (Louis et al., 2015).

With respect to the fact that proteins are synthetized in initial stages of the TE transposition process, it is necessary to realize that proteomics, while allowing for detection of actively transposing TE, also bears some limitations. Feschotte and Pritham (2007) reported that ancient TEs were less likely to be actively transposing, however they might still express proteins, especially when they originated from domesticated TEs, and at present those proteins fulfill essential host cell functions. Altogether, proteomic techniques may provide unique insights to investigations on the TE activity, e.g. involvement of TE-derived proteins in the assembly of protein complexes. However, the employment of complementary strategies is needed to obtain a comprehensive landscape of actively transposing TEs. Proteomics Informed by Transcriptomics (PIT) may be one such prospective strategy. In this method, proteomic MS/MS spectra are searched against open reading frames derived from assembled RNA-Seq transcripts. This approach can reveal previously unknown translated genomic elements or can also identify hotspots of incomplete genome annotation. PIT was initially generated in general principle, however, it can be easily tuned to investigate TE ongoing activity (Davidson et al., 2017; Maringer et al., 2017).

## Detection of extrachromosomal linear DNA

The formation of extrachromosomal linear DNA (eclDNA) molecules is inherent to the process of LTR-RT mobilization. LTR-RTs contain two ORFs, Gag encoding a coat protein, and Pol encoding a polyprotein comprising four domains, i.e. reverse-transcriptase (RT), RNase H (RH), aspartic protease (AP) and integrase (INT). The life cycle of LTR-RTs begins with transcription of an active LTR-RT copy by a host-encoded RNA polymerase II, followed by synthesis of LTR-RT-encoded proteins, formation of virus-like particles (VLPs) encapsulating the RNA template, and its reverse transcription resulting in the formation of eclDNA. Subsequently, eclDNA enters the nucleus and integrates with the host genome (Havecker et al., 2004). Thus, the detection of eclDNAs seems to be an exquisite approach to mine for actively transposing LTR-RTs (Grandbastien, 2015), as they represent the final intermediates in LTR-RT retrotransposition (**Figure 1**). However, eclDNA can occur in cells also as a result of other events, such as cell lysis-originating eclDNA, as cells are constantly being lysed, or extrachromosomal linear microDNA interspersed with microRNAs (Sun et al., 2019). All these eclDNA sources may contain LTR-RT sequences, but only in the case of linear products resulting from the transposition process, the identified fragment is expected to be terminated with LTR sequences, without additional fragments of genomic DNA sequence. Thus, a stage allowing selection of LTR-RTs should be included. A strategy based on PCR amplification utilizing a primer annealing to the tRNA primer binding site (PBS) could be used. It was originally applied to generate PCR-based iPBS molecular markers (Kalendar et al., 2010), while later it became the basis of SIRT (Sequence-Independent Retrotransposon Trapping) – the first method using LTR-RT-derived eclDNAs as targets (Griffiths et al., 2018). It took advantage from the fact that eclDNA ends are blunt-ended and competent for ligation of synthetic adaptors. Subsequently, using PCR primers complementary to the adaptor and to the PBS, a segment comprising the 5´LTR was amplified. When compiling complementary PBS primers, they used the fact that actively transposing LTR-RTs described in plants use predominantly as the initiator methionine tRNA (Met-iCAT) (Wicker et al., 2007; Kalendar et al., 2010). Thus, PBS sequences consist of 12 nucleotides complementary to the terminal nucleotides of the MET-iCAT tRNA. To ensure specific PCR amplification, the PBS-specific primers were therefore extended using the knowledge that two terminal nucleotides of 5′ LTR mostly end in cytidine and adenosine (Griffiths et al., 2018). The disadvantage of the SIRT method is that it utilizes Sanger sequencing and that PBS-anchored primers are specific to particular LTR-RTs, which limits its usefulness for a global analysis of all LTR-RT families. It also turned out that the concept cannot be applied to large and TE-rich genomes,

To eliminate these disadvantages, the ALE-Seq (amplification of LTR of eclDNAs followed by sequencing) approach was developed (Cho et al., 2019). In comparison to SIRT, the ALE-Seq protocol utilizes more versatile primers complementary to PBS (or their combinations), high throughput sequencing, and is more elaborate as it includes adapter ligation, transcription and reverse transcription targeted to PBS domains. On the other hand, the ALE-Seq protocol is markedly more selective and efficient than SIRT, which relies on the single PCR amplification (Cho et al., 2019). The method is relatively recent, its applicability has been proved by the identification of actively transposing LTR-RTs in rice and tomato. On the basis of subsequent clustering of sequenced reads some retroelements were recognized as newly identified families for the respective genome. To summarize, ALE-Seq has potential for future use allowing reference-free annotation of new, active retroelements, what is especially important in plant species for which no reference genome assemblies are available (Satheesh et al., 2021).

## Detection of extrachromosomal circular DNA

Some LTR-RT-derived eclDNA molecules were shown to be circularized. As integrase (IN) molecules are attached to LTRs of eclDNAs, their homodimerization causes the formation of a pseudocircular but unclosed structures. Following their recognition as double strand breaks by DNA repair machineries in the nucleus, they are ligated resulting in closed extrachromosomal circular DNA (eccDNA) molecules (**Figure 1**). As such, they do not directly participate in the process of transposition and can be seen as mobilization by-products, however, their presence provides information about actively transposing LTR-RTs (Lanciano et al., 2017).

It should be stressed LTR-RT transposition is not a sole source of eccDNAs; they can also occur as a result of other cellular processes. They are common in eukaryotes and can be very heterogenic in number, length, origin, and role as reviewed by Cao et al. (2021).

The first methods of eccDNA detection, i.e. inverse PCR amplification of LTR-LTR junctions and electron microscopy, suggested that some circles originated from TEs, mostly LTR-RTs (Hirochika and Otsuki, 1995) and Mutator-like class II elements (Sundaresan and Freeling, 1987). Advances in sequencing techniques contributed to the development of efficient eccDNA detection methods along with the bioinformatics tools for analysis of such data.

The first high-throughput method of sequencing eccDNA, Circle-Seq, was developed for yeast and consisted of alkaline-based extraction of circular DNA, followed by digestion of linear DNA, eccDNA amplification using φ29 DNA polymerase and sequencing on the Illumina platform using SE mode (Møller et al., 2015). Soon after, based on similar assumptions, a standardized Mobilome-seq protocol of extraction and Illumina SE sequencing of eccDNA from plant tissues was established (Lanciano et al., 2017). Another approach, CIDER-Seq (Circular DNA Enrichment sequencing) method, originally developed for analysis of plants infected with viruses, utilizes electrophoresis-based size-selection as the first step of sample preparation, followed by random amplification of circular DNA with φ29 DNA polymerase, repair by DNA polymerase I and sequencing using Single Molecule Real Time sequencing (Pacific Biosciences) (Mehta et al., 2019).

The production of large amounts of sequencing data raises the need for simultaneous development of analytical tools. Circle-Map (Prada-Luengo et al., 2019) and Circle_finder (Kumar et al., 2020) were developed for identification of human tumor related eccDNA sequenced using short-reads technology. The downside to these tools is that they both require a reference genome as an input file and they were not tested on plant data. Short reads can be also analysed using ECCsplorer (Mann et al., 2022), a tool for mapping reads to the reference genome, identifying genomic origin of eccDNAs on the basis of read distribution, coverage, discordant mapping, and split reads, but also enabling reference-free clustering of reads. This helps to identify and annotate LTR-RTs enriched in eccDNA libraries. eccDNA analysis from long reads is possible using the CIDER-seq2 (Mehta et al., 2020). Although the method was developed for identification and characterization of plant virus genomes, and includes the ‘annotate’ module that is restricted to viruses annotation, part of the pipeline that outputs eccDNA candidates and their genomic localization can be used for the identification of LTR-RTs. Other long-reads based tools, such as CReCIL (Wanchai et al., 2022) allow not only efficient identification of circular DNA but also annotation and Circos-based visualization of assembled circles, but its performance was tested only on long-reads from mammals eccDNA sequencing. Another tool, ecc_finder (Zhang et al., 2021) is based on a pipeline applied for the analysis of Mobilome-seq data originated from plant tissues (Lanciano et al., 2017). The pipeline allows analysis of both short and long reads and can be run in the reference genome and reference-free modes.

The eccDNA identification was reported to be useful for monitoring mobilization of previously known actively transposing TEs in Arabidopsis, rice and tomato (Lanciano et al., 2017; Benoit et al., 2019; Lanciano et al., 2021; Roquis et al., 2021; Wang et al., 2021; Zhang et al., 2021; Mann et al., 2022) and *de novo* identification of mobilized LTR-RTs, as shown for potato (Esposito et al., 2019), poplar (Sow et al., 2021) and carrot (Kwolek et al., 2022).

Mapping eclDNA or eccDNA sequencing reads to the reference genome may provide a clue as to what is the TE copy that has been undergoing mobilization. Ideally, a reference genome highly related to the individual used for eclDNA or eccDNA should be used. However, the typical properties of TEs, such as their highly repetitive character and the fact that TE families can be highly interrelated within a given species may complicate conclusions driven from such analyses.

## Identification of novel insertion sites produced by actively transposing elements

The life cycle of a TE is completed upon its insertion into a new position in the host genome (**Figure 1**). Such *de novo* insertions are thus present in the progeny while they are absent in the ancestral plants. In earlier studies, these uncommon events were recognized only when they resulted in changed phenotypes. Obviously, these events represent a very small proportion of the total number of successful transpositions resulting in the integration occurring in genic regions.

Historically, the principles of positional (genetic map-based) cloning were used to identify insertional polymorphisms in the genome. However, mapping with high resolution requires numerous mapping populations and many genetic markers, thus it is costly and time consuming. It is therefore not suitable for mapping newly transposed TEs, although one can find some examples here as well (Bortiri et al., 2006). Identification of TE insertion sites and resulting transposon insertion polymorphisms (TIPs) can be also performed using marker systems derived from conservative sequences specific to certain TEs (Kalendar and Schulman, 2006) or by a modification of the amplified fragment length polymorphism (AFLP) protocol (Vos et al., 1995). It is based on comparing the distribution of copies of a particular TE family in a collection of closely related accessions and works especially well for TE families with a number of copies highly uniform in their sequence, which is a proxy for recent or ongoing transposition. Two AFLP modifications aiming at the identification of TIPs have been developed, i.e. sequence-specific amplification polymorphism (S-SAP), used for the identification of LTR-RT insertions, where the final amplification is performed with a retrotransposon-specific and a *Mse*I-adaptor-specific primer (Waugh et al., 1997), and transposon display (TD) using two rounds of PCR with nested transposon-specific primers (Casa et al., 2000; Grzebelus et al., 2007) and applied mostly to identify TIPs produced by DNA transposons. Those methods have often been used to identify TIPs derived from few known TE families. One of the first attempts where the S-SAP method was successfully applied to identify a newly inserted LTR-RT was reported by Tahara et al. (2004). They identified Ty1-copia retrotransposons in sweet potato activated in the callus. Similar approach was used by Yamashita and Tahara (2006), where a polymorphic S-SAP product was identified as a LINE retroelement activated in meristem stem cells. There are examples of S-SAP being successfully used also to identify ongoing transpositions upon stress other than *in vitro* cultures. For example, Woodrow et al. (2010) identified Ty1-copia transposition in durum wheat under salt and light stress. The effect of interspecific hybridization and polyploidization on the actively transposing LTR-RT using S-SAP was evaluated by Gantuz et al. (2022). Another TIP identification system named palindromic sequence-targeted PCR (PST-PCR v.2) was proposed by Kalendar et al. (2021). It relies on the use of capturing primers targeting palindromic sequences arbitrarily present in natural DNA templates in combination with a sequence –specific primer. PST-PCR v.2 consists of two rounds of PCR. The first round utilizes a combination of one sequence-specific primer with one capturing (PST) primer. The second round uses a combination of a single (preferred) or two universal primers; one anneals to a 5′ tail attached to the sequence-specific primer and the other anneals to a different 5′ tail attached to the PST primer. The key advantage of PST-PCR v.2 is to quickly produce amplified PCR fragments containing a portion of the template flanked by the sequence-specific and capturing primers. The approach allowed characterization of Ac transposon integration sites (Kalendar et al., 2021). Lack of restriction digestion and adapter ligation, i.e. steps required in S-SAP or TD, reduces the cost and time of identifying new insertion sites.

All wet-lab methods are primarily useful for monitoring the mobilization of previously identified TEs, e.g. under stress conditions or in a range of genetically diverse accessions, since they require the use of primers with a sequence specific to the sequence of the investigated TE. Moreover, the specificity of the amplification and the reliability of the new insertion sites should be confirmed by sequencing.

In 2004, the 454 technology became commercially available next generation sequencing (NGS) platform. Since then, NGS began to be widely applied to study plant TEs. In the early stages, they were usually combined with other techniques based on PCR amplification of regions specific to TEs. As an example, Monden et al. (2014), produced a LTR-RT libraries derived from eight strawberry cultivars, based on the primer binding site (PBS) adjacent to the conserved 5′ LTR motif and sequenced them using Illumina HiSeq2000. It allowed detection of cultivar-specific LTR-RT insertion sites.

Another approach for genome-wide TIPs detection produced by a single TE family includes AFLP-based enrichment of DNA fragments in TE sequences followed by Illumina library preparation and sequencing. The recently published TEAseq pipeline (Lyu et al., 2021) developed for maize Ds transposons consists of samples barcoding, TE enrichment, library preparation and Illumina sequencing. The bioinformatics workflow for sequencing data analysis starts from de-barcoding, next reads containing the TE sequence are identified, the TE-portion of the read is trimmed and the remaining portion of the sequence is mapped against the reference genome to identify the insertion site. The method was successfully used for the identification of 35,696 putative germinal insertion sites in over 1,600 Ds insertional mutants. The major advantage of such approach is not only more detailed information about the number of TE insertions and the level of polymorphism among tested individuals but also the availability of sequences of regions flanking insertions, that is vital for verification of novel insertion sites and their downstream analyses.

With the advent of high throughput sequencing technologies, strategies have been developed to mine for TE insertion sites using raw reads and a suite of bioinformatics tools is currently available (Serrato-Capuchina and Matute, 2018; Vendrell-Mir et al., 2019; Fan et al., 2022). Depending on the purpose of the analysis and the type of investigated TEs, different tools and approaches are being developed. Some tools like the TRACKPOSON (Carpentier et al., 2019) can identify TIPs very quickly and efficiently using discordant reads identified in the process of reads mapping against a TE sequence for the identification of insertions based on their position in the reference genome. It shortens time of the analysis at the expense of the precise determination of the site of insertion. Nevertheless, the identification of ‘insertion signatures’, i.e. TE sequences in specified genomic windows rather than their precise locations, might be the first choice for large-scale analysis of LTR-RTs, including thousands of re-sequenced genomes, as shown for the analysis of 3,000 rice genomes (Carpentier et al., 2019). The method reports both reference and non-reference insertions and does not require any prior TE annotation in the reference genome.

Tools based on the usage of discordant reads and split-reads report precise localization of insertion sites. That group of tools often requires high quality annotation of TEs in the reference genome, which in case of non-model organisms may limit their utility. In spite of higher computation demands, they can be efficiently used for large-scale population studies. Evaluation of this type of analysis make easier if another selective step is included in the experiment, such as the principle of TE sequence capture described firstly by Baillie et al. (2011) on the example of retrotranspositions registered in the human brain. Subsequently, this principle was used by Quadrana et al. (2016) in mining of transposition events within sequencing data for 211 *Arabidopsis thaliana* accessions. The SPLITREADER used here (Quadrana et al., 2016) was utilised for a global analysis of LTR-RTs in 602 tomato accessions and TIP-based GWAS (TE-GWAS; TIP-GWAS), that allowed identification of retrotransposon insertions associated with important phenotypic traits, such as flavor (Domínguez et al., 2020), while insertional polymorphism of class II MITEs in 3,000 rice genomes was analysed using PoPoolationTE2 (Kofler et al., 2016) and TIP-based GWAS showed association of particular MITE copies with MITE copy number, suggesting that MITE subfamilies originate from few “master” copies (Castanera et al., 2021). Another short read based method, RelocaTE2 (Chen et al., 2017) was used to analyse copy number and distribution of mPing, Ping and Pong class II elements actively transposing in rice in 3,000 rice genomes (Chen et al., 2019) and to detect *de novo* insertions of mPing in 272 rice recombinant inbred lines (RILs) developed from a cross between Nipponbare and HEG4 known to carry active mPing (Chen et al., 2020).

The obvious prerequisite for their utilization is availability of a high quality reference genome. The combination of high throughput sequencing and *in silico* discovery of new TE insertion events currently seems to be the most efficient strategy. Nevertheless, the risk that some new insertions are not being recorded still remains, but can be reduced by sufficient amount of reads i.e. it is necessary to achieve a high sequencing coverage.

It is also possible to utilize a pan-genome approach, i.e. to compare two or more genome assemblies representing the same or closely related species, with the intention of finding TIPs differentiating those genomes. However, availability of multiple genome assemblies limits the usage of such approach to the identification of TIPs and analyses of contribution of TEs to genome organization, as shown for four maize genotypes (Anderson et al., 2019), rather than for tracking or identification of active TEs.

A further significant improvement in the identification of TIPs may be achieved by the use of long-read NGS techniques, such as the Oxford Nanopore technology (ONT) (Ellison and Cao, 2020; Ewing et al., 2020). While short reads technologies work well in identifying insertion sites of small TEs, such as MITEs, long reads significantly improve the efficiency of analysis of longer elements, especially LTR-RTs that are the most abundant TEs in plant genomes. For example, the utility ONT was shown for detection of novel insertions of actively transposing LTR-RTs in Arabidopsis; EVD (Debladis et al., 2017) and ONSEN (Kirov et al., 2021), as well as for the identification of TIPs in collections of insertional mutants of Medicago and soybean (Song et al., 2021). Along with the development of long read sequencing, tools dedicated to the identification of insertions in such data are becoming available. The first tool identifying TIPs in long read data was PALMER (Pre-mAsking Long reads for Mobile Element insertion), based on the alignment of reads to the genome and masking reference insertions of the investigated TE family in the reads sequence. Subsequently, the TE sequence is identified in the unmasked part of the read and, based on the presence of specific features, the software identifies ends of TE, and the remaining part of the read is used to detect non-reference insertion sites (Zhou et al., 2020). The method was successfully applied to the human genome and it was adjusted to the most common human TEs (L1, Alu, SVA). Hence it may not work for other types of TEs, e.g. those abundant in plant genomes. Another pipeline, also developed to screen actively transposing human TEs, utilizes a slightly different strategy, as in the reads the portion mapped to the genome is masked, while the remaining part is mapped to a TE library, TE sequences are reconstructed and the remaining part of the sequence is re-mapped to the reference genome to identify non-reference insertions. In addition to TIPs identification, this pipeline allows analysis of TEs methylation, that is called by the software dedicated for identification of CpG methylation in ONT reads (Ewing et al., 2020). The long read sequencing methods produce reads overlapping full TE sequences and their flanking regions, providing opportunity for comprehensive characterization of those sequences. They also allow identification of TEs insertions within repetitive regions. However, for the identification of novel insertions of actively transposing elements, especially in plants, the Illumina platform is still a method of choice, as efficient bioinformatic tools have been available and the cost of sequencing is still much lower. The Cas9-targeted sequence capture to enrich library with TE sequences, in combination with long read sequencing, may be an alternative solution, that would reduce the cost of sequencing while still benefiting from the advantage provided by long reads (McDonald et al., 2021).

Long read sequencing also improves genome assemblies in TE-rich regions, TE detection, annotation and identification of TIPs (Shahid and Slotkin, 2020), opening new perspectives for better understanding of the TE biology and activity.

**Figure 2:**
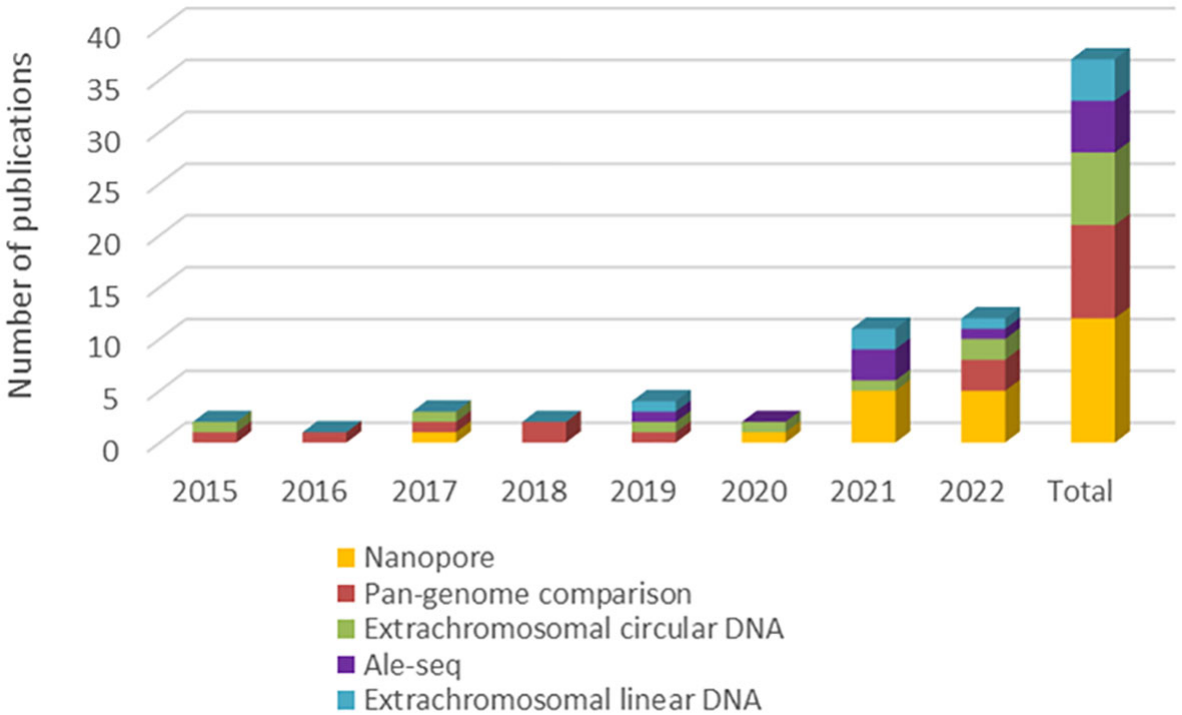
Popularity estimation of selected perspective approaches based on the frequency of their use in recent scientific articles. * The number of publications was generated by a search combining core keywords “plant + transposable + activ*” and keywords corresponding to individual perspective approaches.

Based on the information provided, a screening was carried out to estimate the popularity of selected perspective approaches in the last period (see **Figure 2**). Here it is confirmed that the frequency of their use is generally increasing, especially in the last 2 years, while the use of Oxford Nanopore technology seems to be as most frequently used from compared approaches. Finally, the most important advantages and disadvantages of all discussed detection techniques were summarized (see **Table 1**).

**Table 1:** Summary of approaches used to identify actively transposing elements.

Strategy used to identify actively transposing TEs	Main drawbacks	Recommendations for efficient targeting actively transposing TEs
Targeting TE-derived transcripts	- existence of TE-derived transcripts not competent for transposition (chimeric transcripts; transcripts involved in epigenetic silencing of TEs; post-transcriptional suppression mechanisms by TE-derived sequences)	- combine with another technique targeting products from the final phases of the transposition process (e.g. eclDNA, eccDNA)
Targeting TE-derived proteins	- non-transposing TEs can still express proteins - requires equipment that is not so common in molecular genetics laboratories	- combine with another technique targeting products from the final phases of the transposition process (e.g. eclDNA, eccDNA) - PIT (Proteomics Informed by Transcriptomics)
Targeting eclDNA	- eclDNA can occur as a result of other cellular processes (e.g. cell lysis, existence of micro-eclDNA)	- include a selective step to enrich TE-derived eclDNAs (e.g. PBS complementary to MET-iCAT tRNA) - combine with high throughput sequencing (ALE-Seq)
Targeting eccDNA	- eccDNA does not directly participate in the process of transposition - eccDNA can occur as a result of other cellular processes	- combine with high throughput sequencing to identify novel insertion sites
Identification of novel insertion sites by using TE-based genotyping platforms	- laborious and time consuming and error-prone	- use PST-PCR v.2 as a less laborious method
High throughput sequencing	- availability of a high quality reference genome or a large set of resequenced genomes of related accessions - inaccuracies related to short reads provided by the Illumina technology (problems with longer TEs, such as LTR-RTs; insertions in repetitive regions)	- use technologies producing long reads, e.g. Oxford Nanopore

## Concluding remarks and future perspectives

Historically, the importance of TEs in plant genomes has been neglected. However, it turned out that their presence affects many areas important for the life and development of plants, as well as in terms of their possible use in the field of plant breeding. It puts pressure on the availability of suitable analytical methods to trace the pathways of actively transposing TEs. However, the interpretation of results produced by the above-presented methods can be difficult owing to the inherent properties of TEs. This review seeks to present techniques that can be used to obtain information about mobilized TEs and some pitfalls associated with the interpretation of results. The methods were divided on the basis of the context of their use with respect to the process of transposition.

Apparently, the use of some of the older methods mentioned above can be expedient in some specific cases and can bring unique information at relatively low price and experimental demands. The most comprehensive results are seemingly achievable by the methods based on massive parallel sequencing, however, they have also their limits. One such limitation is the fact that the created evaluation tools detect only a limited part of TEs. Related to this is also the need for thorough genomic TE annotation as an important prerequisite for appropriate detection of new copies. Some of shortcomings in the accuracy in bioinformatics data interpretation can be significantly improved by NGS techniques producing long reads. Generally, the strengths of one method are usually offset by other shortcomings. To obtain a comprehensive picture, a combination of methods based on different principles, seems to be the most effective. One of such examples is a strategy combining RNA-seq and MS, for which the designation Proteomics Informed by Transcriptomics is used. From the principle of the matter, a combination of methods targeting molecules originating from the final stages of the transposition process of actively transposing TEs seems to be the most suitable. Namely, it means to focus on methods aimed at detecting novel insertion sites, eclDNA and eccDNA. From this perspective, coupling WGS and analysis of the intermediates or by-signals of actively transposing TEs, such as eccDNA, ALE-Seq or multi-genomic comparisons, seems to be a promising approach to reveal complete information regarding TEs activity and their impact on host genome.

## Author contributions

MBaj wrote first draft of the manuscript and perform graphical support. AP wrote sections of the manuscript that referred to current bioinformatics tools. DG contributed to conception, compiled and revised author contributions. MBar established conception and design, wrote some parts of manuscript and revised author contributions. All authors contributed to the article and approved the submitted version.

## Funding

This research was funded by Internal Grant of Mendel University (IGA-ZF/2021-SI1007) and by the project CZ.02.2.69/0.0/0.0/16_018/0002333 Research Infrastructure for Young Scientists, this is co-financed from Operational Programme Research, Development and Education.

## Conflict of interest

The authors declare that the research was conducted in the absence of any commercial or financial relationships that could be construed as a potential conflict of interest.

## Publisher’s note

All claims expressed in this article are solely those of the authors and do not necessarily represent those of their affiliated organizations, or those of the publisher, the editors and the reviewers. Any product that may be evaluated in this article, or claim that may be made by its manufacturer, is not guaranteed or endorsed by the publisher.
